# Effectiveness of a structured, framework-based approach to implementation: the Researching Effective Approaches to Cleaning in Hospitals (REACH) Trial

**DOI:** 10.1186/s13756-020-0694-0

**Published:** 2020-02-18

**Authors:** Lisa Hall, Nicole M. White, Michelle Allen, Alison Farrington, Brett G. Mitchell, Katie Page, Kate Halton, Thomas V. Riley, Christian A. Gericke, Nicholas Graves, Anne Gardner

**Affiliations:** 1 School of Public Health, University of Queensland, 288 Herston Road, Herston, Queensland 4006 Australia; 2 School of Public Health and Social Work, Queensland University of Technology, Brisbane, Queensland Australia; 3 Australian Centre for Health Service Innovation, Institute of Health and Biomedical Innovation, Queensland University of Technology, Brisbane, Queensland Australia; 4 Abt Associates, Brisbane, Queensland Australia; 5 Discipline of Nursing, Avondale College of Higher Education, Cooranbong, New South Wales Australia; 6 School of Nursing and Midwifery, University of Newcastle, Newcastle, New South Wales Australia; 7 Centre for Health Economics Research and Evaluation, University of Technology Sydney, Ultimo, New South Wales Australia; 8 School of Biomedical Sciences, University of Western Australia, Perth, Western Australia Australia; 9 School of Medical and Health Sciences, Edith Cowan University, Perth, Western Australia Australia; 10 School of Veterinary and Life Sciences, Murdoch University, Perth, Western Australia Australia; 11 School of Clinical Medicine, University of Queensland, Brisbane, Queensland Australia; 12 School of Nursing, Queensland University of Technology, Brisbane, Queensland Australia

**Keywords:** Environmental cleaning, Implementation science, Infection prevention

## Abstract

**Background:**

Implementing sustainable practice change in hospital cleaning has proven to be an ongoing challenge in reducing healthcare associated infections. The purpose of this study was to develop a reliable framework-based approach to implement and quantitatively evaluate the implementation of evidence-based practice change in hospital cleaning.

**Design/methods:**

The Researching Effective Approaches to Cleaning in Hospitals (REACH) trial was a pragmatic, stepped-wedge randomised trial of an environmental cleaning bundle implemented in 11 Australian hospitals from 2016 to 2017. Using a structured multi-step approach, we adapted the integrated Promoting Action on Research Implementation in Health Services (i-PARIHS) framework to support rigorous and tailored implementation of the cleaning bundle intervention in eleven diverse and complex settings. To evaluate the effectiveness of this strategy we examined post-intervention cleaning bundle alignment calculated as a score (an implementation measure) and cleaning performance audit data collected using ultraviolet (UV) gel markers (an outcome measure).

**Results:**

We successfully implemented the bundle and observed improvements in cleaning practice and performance, regardless of hospital size, intervention duration and contextual issues such as staff and organisational readiness at baseline. There was a positive association between bundle alignment scores and cleaning performance at baseline. This diminished over the duration of the intervention, as hospitals with lower baseline scores were able to implement practice change successfully.

**Conclusion:**

Using a structured framework-based approach allows for pragmatic and successful implementation of clinical trials across diverse settings, and assists with quantitative evaluation of practice change.

**Trial registration:**

Australia New Zealand Clinical Trial Registry ACTRN12615000325505, registered on 4 September 2015.

## Contributions to the literature

- There is growing evidence of the importance of a clean hospital environment in reducing transmission of healthcare associated infections, however little is known about how to implement improvements in hospital cleaning in a pragmatic and sustainable way. In a stepped-wedge randomised trial (published elsewhere), we showed that an environmental cleaning bundle was effective at reducing infections, and was cost-effective to implement.
- For this trial we used the integrated Promoting Action on Research Implementation in Health Services (i-PARIHS) framework to design an implementation strategy. This included developing a novel set of templates for context mapping and guiding the implementation process of the bundle. We also used these templates to quantitatively evaluate the extent of implementation.
- The current study focusses on how a framework-based approach allowed us to balance the priorities of a rigorous protocol driven clinical trial with the need for flexible local tailoring to improve uptake and fidelity of the intervention in eleven very different hospitals.
- Although it is well known that the use of a framework can facilitate implementation of an intervention in a quality improvement setting, this approach has been rarely used in infection prevention clinical trials, and had not been applied to environmental cleaning before this study.
- We showed this approach was successful in improving implementation of the intervention and resulted in improvements in cleaning performance.

## Introduction

Hospital cleaning is complex [1]. Implementing sustainable practice change in this area has proven to be an ongoing challenge in reducing healthcare-associated infections (HAIs) internationally. There is increasing evidence of the importance the hospital environment plays in the transmission of infections [2], however, there is disagreement on how best to improve cleaning [3] and the “wicked problem” [4] of implementation. Once we know what to do, how can we maximise the chances of best practice being used, in a pragmatic and sustainable way?

The field of implementation science helps to bridge this gap by providing a systematic focus on what helps and hinders uptake, effective implementation and sustainability of practice [5]. The use of implementation science in infection prevention is a rapidly changing field. Great advances have been recently made by the *On the CUSP-Stop CAUTI* and the *Michigan Keystone* projects which have successfully employed a multifaceted structured approach to the national scale-up of quality improvement programs [6, 7]. However, implementation science frameworks have been rarely used in clinical trials evaluating infection prevention interventions. At the commencement of our research, these frameworks had not been applied to address environmental cleaning in hospitals.

In the Researching Effective Approaches to Cleaning in Hospitals (REACH) trial we sought to distil the key evidence-based practices around cleaning and develop a reliable framework-based approach to implement and quantitatively evaluate the implementation of practice change [8, 9]. Our pragmatic approach accounted for variation in context, and the existing evidence-practice gaps across trial sites. We had to address two potentially opposing perspectives ─ that of the clinical researcher, requiring adherence to protocol and academic rigour in design and analysis, balanced with that of participants who required a real-world intervention and implementation approach that was flexible, acceptable, useful and empowering to staff.

We have demonstrated in previous analyses that the REACH intervention was effective at reducing healthcare associated infections and cost-effective to implement in hospitals [9, 10]. This paper describes the development and application of the implementation strategy employed in the REACH trial, and aims to evaluate the effectiveness of this framework based approach using two quantitative measures of implementation success: post-intervention cleaning bundle alignment (an implementation measure), and cleaning performance (an outcome measure).

## Methods

### Study design and setting

The REACH trial was a pragmatic, stepped-wedge randomised trial of an environmental cleaning bundle implemented in 11 Australian hospitals from 2016 to 2017. Eligible hospitals were recruited as per the study protocol [8], with nine public hospitals and two private hospitals enrolled in four of the eight Australian states and territories. Across the 11 hospitals, the mean number of hospital overnight beds was 500 (range 227 to 930). Within each hospital, a cleaning intervention was implemented in collaboration with environmental services staff who had a role in ward cleaning.

### Intervention

The REACH environmental cleaning bundle was developed through a structured search and review of the literature, followed by an expert panel process that identified and then prioritised evidence-based strategies for inclusion in the bundle. Feasibility and cost of implementation, as well as effectiveness, were considered when choosing bundle components. Best practice processes for staff training, communication and audit were included, in addition to specifications around cleaning technique and type of products to use. The bundle was piloted at a large Brisbane hospital in 2014, with promising results [11].

The cleaning bundle consisted of five components [12]. These were:

- Training – Training sessions were delivered to environmental services teams and included content on the impact of environmental cleaning on HAIs, cleaning roles and responsibilities, and instructions on how to use the REACH cleaning bundle.
- Technique – This emphasised the importance of a defined and consistent cleaning sequence, daily cleaning of the high risk frequent-touch points (FTPs) and the use of sufficient pressure and movement.
- Product – This required use of a disinfectant for all discharge cleans and for daily cleans of high risk/precautions rooms; use of detergent for routine cleans; use of point-of-care wipes for medical equipment, and adherence to manufacturers’ instructions for all product use.
- Audit – This involved monthly audit activities at each hospital using ultraviolet (UV) fluorescent marker technology. The gel markers leave dots that are invisible to the naked eye, but are removed completely by routine cleaning. Trained hospital staff applied gel dots to FTPs in patient bedrooms and bathrooms in randomly selected Intensive Care Unit (ICU) and general ward rooms [13, 14]. FTPs were checked after cleaning for removal of the gel dots. Staff received individual feedback about audit results. Summarised audit results were also provided to environmental services teams and to hospital clinical governance committees.
- Communication – This included promotional activities to raise the profile and importance of environmental services staff and their work. It emphasised and encouraged daily contact between cleaning staff and ward leaders, and the inclusion of cleaning staff representatives on relevant clinical governance committees.

### Implementation strategy

To support effective implementation of a complex intervention into 11 diverse hospital settings, the REACH trial used an implementation science approach, specifically the integrated Promoting Action on Research Implementation in Health Services (i-PARIHS) framework [15]. Using a structured multi-step approach, we adapted this framework to support rigorous and tailored implementation of the cleaning bundle intervention. The framework facilitated regular monitoring and documentation of the implementation process, as well as fidelity of the cleaning bundle implementation. Understanding the process of implementation and knowledge of what worked where and in what conditions was important for trial site comparisons, as well as informing future replication and scalability.

We first developed an intervention logic model (Additional file 1) – a pictorial representation of key trial inputs, outputs and outcomes – to clarify the underlying assumptions about the hospital environmental cleaning change process [16]. The iPARIHS framework was then used to guide the development of an implementation toolkit. This toolkit: (1) provided structure for the systematic collection and assessment of initial contextual information at each site using a series of templates and tools, (2) informed the tailoring of the cleaning bundle intervention and development of a site-specific implementation plan at each site, and, (3) guided the ongoing monitoring and documentation of the implementation process at each site and concurrent changes to local context.

The implementation toolkit contained:

- A detailed description of the intervention that documented the essential, fixed and flexible elements of each bundle component.
- The REACH implementation framework, created by mapping the iPARIHS constructs of intervention characteristics, intervention recipients and context (inner local, inner organisation, and outer context) to the REACH cleaning bundle (Additional file 2).
- A series of templates to inform the operationalisation of the framework and provide rigour for each stage of implementation. Templates guided systematic assessment of each hospital’s baseline practices (document review, hospital profile), quantification of the evidence-practice gap in relation to each bundle component, and the pre-trial identification of contextual barriers and enablers at each site. We also developed templates for site implementation plans, and for monitoring and evaluating the implementation process.

### Project governance

A decentralised model of facilitation was employed in this project. Overall governance was provided by the Management Committee – comprising the research investigators who designed the study. The study team was comprised of a small group of trained researchers, who carried out the context mapping, worked with the hospitals to tailor the implementation strategy and roll-out the trial. The study team was led by the project manager and reported to the management committee. At each hospital there was a small team of staff members (the site team) who were nominated as a point of contact for the study team, and responsible for enacting the implementation plan, and ongoing data collection. Members of the site team were trained in gel dot auditing and feedback by the study team.

### Quantifying implementation

At each hospital during the pre-intervention period, the study team collected extensive baseline data about infection prevention and hospital cleaning using staff questionnaires [17], interviews, discussion groups, and the document review and hospital profile templates. For each hospital, contextual data were then systematically mapped against the implementation framework (additional file 2) and assessed to determine three separate sets of scores.

The first set of scores related to: alignment of current practice against the five bundle components (iPARIHS intervention characteristics). The second set referred to staff readiness (iPARIHS intervention recipients) and included ratings for motivation to change, capacity to change, resources, and support. The third set examined site implementation readiness (iPARIHS context) including inner local context, inner organisational context and outer context.

**Fig. 1:**
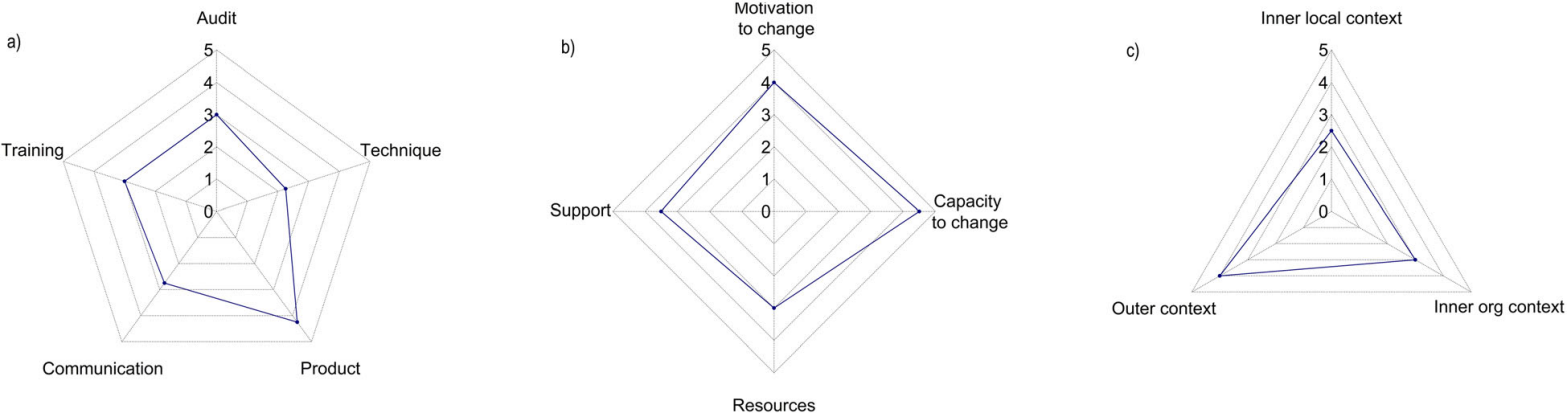
Example mapping of pre-intervention: (**a**) bundle alignment; (**b**) staff readiness; (**c**) site readiness for implementation

To determine these scores, two members of the study team independently reviewed the context mapping information and rated all items on a 0 (low) -5 (high) scale. They then reviewed the scoring and contextual information together, discussed discrepancies, and agreed a final score. On the few occasions where consensus could not be reached, a midpoint was chosen. Ratings were summarised on a series of web diagrams for each hospital (Fig. 1).

Quantifying evidence-practice alignment and the extent of contextual enablers at each hospital provided not only a score but also a visual picture of gaps and barriers to be addressed. This informed development of a tailored site-specific implementation plan and supported the pragmatic roll-out of the bundle. Each plan included detailed information about activities, timings and responsibilities for effective implementation.

Baseline information and webs were also used as a reference point for monitoring implementation progress. Throughout the trial, the study team maintained detailed notes on all contact with each hospital. The team completed training reports, a two-monthly monitoring survey with the hospital site team, post-trial questionnaires with environmental services staff and close-out meetings with each site team. Post-trial we systematically reviewed this information and made comparisons with information in the pre-trial records and the agreed implementation plan to assess the extent of implementation of each bundle component. Items were again rated using the same 0–5 scale for each component and recorded on a five point bundle alignment web.

### Measuring cleaning performance

As a measure of cleaning performance, gel dot audit data were collected and submitted by trained auditors at each hospital. This included information on the location and date of each FTP audited, and whether the FTP was deemed cleaned based on removal of the gel dot [8, 18].

### Data analysis

Descriptive statistics summarised bundle alignment scores recorded in the pre-intervention and intervention periods by bundle component. Analysis initially focused on all hospitals, with score changes expressed as mean differences. Additional analysis considered the number of hospitals with the potential to improve their alignment in the intervention period, and the subsequent number of hospitals that improved. For each bundle component, hospitals with a pre-intervention alignment score of 0–4 were defined as having potential to improve following implementation.

Changes in bundle alignment scores were further compared by baseline hospital characteristics, including: the number of overnight beds (≤350, 351–600, > 600); intervention duration (1–30 weeks, 31–50 weeks); cleaning workforce (single, dual); pre-intervention staff readiness and site implementation readiness scores. Mean differences by hospital characteristic were tested using one-way analysis of variance.

To examine associations between trial implementation and effectiveness outcomes, pre-intervention alignment scores were compared with the results of monthly audit activities. Monthly audit outcomes collected during the pre-intervention and intervention periods were summarised by the number of FTPs audited and the number of FTPs successfully cleaned. Data were analysed using a binomial mixed model, which described changes in the proportion of FTPs cleaned. The mixed model included a random effect for each hospital, and fixed effects for pre-intervention alignment score and the timing of each audit in weeks since the start of the intervention period. A two-way interaction term was also included to test if changes in cleaning performance over time were influenced by pre-intervention bundle alignment.

Model outputs were reported as odds ratios with hypothesis testing of effects based on a 5% level of statistical significance. Model-based predictions of cleaning performance and confidence intervals (CI) were calculated using a parametric bootstrap. Interaction effects were summarised graphically to compare trends in cleaning performance between hospitals with low, average and high alignment, with categories based on the observed range of total pre-intervention scores. All analyses were conducted in R 3.5.1 [19]. Study findings are reported in line with the Standards for Reporting Implementation Studies (StaRI) checklist [20].

## Results

### Implementation measure bundle alignment scores

Prior to implementation, total bundle alignment scores ranged between 9.5 and 20 with an average score of 15 (95% CI: 13.4 to 16.7). Overall, the mean total alignment score improved by 3.5 points (95% CI: 2.0 to 5.0, *p*-value: 0.0003) to 18.5 points in the intervention period (95% CI: 17.0 to 20.0).

At baseline, all 11 hospitals had at least one bundle component requiring improvement. We examined improvements observed by bundle component (Table 1). The greatest improvement in mean bundle alignment score across all hospitals was observed for the audit component (2.6 to 3.6). When we examined the number and percentage of hospitals who improved after implementation the biggest changes were observed for the technique (75%) and training (56%) components.

**Table 1:** Summary of improvements in alignment by bundle component

Bundle component	Bundle alignment score Mean (SD)		Hospitals with potential to improve alignment	Hospitals that improved after implementation
	Pre	Post	N (%)	N (%)
Audit	2.6 (0.69)	3.6 (0.98)	10 (91)	5 (50)
Communication	2.9 (0.89)	3.5 (0.79)	9 (82)	3 (33)
Product	3.5 (1.20)	4.0 (0.90)	6 (55)	3 (50)
Technique	3.4 (0.81)	3.9 (0.57)	8 (73)	6 (75)
Training	2.7 (0.94)	3.5 (0.73)	9 (82)	5 (56)

*SD* Standard deviation, *N* Number of hospitals

The smallest improvement was for communication (33%). Our qualitative records showed that in some trial hospitals miscommunication and misunderstanding occurred, primarily in hospitals without a locally based facilitator.

Increases in bundle alignment were seen regardless of baseline hospital characteristics (Table 2). Hospital size, staff and organisational (site) readiness at baseline, type of cleaning workforce and the duration of intervention were not associated with pre-alignment scores and were not associated with overall changes in bundle alignment.

**Table 2:** Hospital characteristics at baseline, and association with total bundle alignment scores before and after implementation

Characteristic	Level	N	Bundle alignment score Mean (SD)			*p*-value	
			Pre	Post	Difference	Pre	Difference
Hospital size (Number of overnight beds)	<=350	3	16.0 (0.75)	19.1 (0.55)	3.1 (0.86)	0.77	0.90
	351–600	4	15.0 (1.24)	18.5 (2.25)	3.4 (2.38)		
	600+	4	14.3 (4.68)	18.2 (3.85)	3.9 (3.03)		
Cleaning workforce	Single		15.3 (1.57)	19.0 (2.49)	3.7 (2.28)	0.70	0.68
	Dual		14.6 (4.47)	17.7 (2.56)	3.1 (2.24)		
Duration of intervention (weeks)	1–30	4	16.0 (3.40)	18.7 (3.35)	2.7 (3.45)	0.42	0.37
	31–50	7	14.5 (2.45)	18.5 (2.15)	4.0 (1.10)		
Staff readiness score	5–9.99	1	9.5 (−)	14.4(−)	–	–	–
	10–14.99	7	14.9 (1.67)	18.7 (2.52)	3.8 (2.19)		
	15–20	3	17.3 (2.54)	19.7 (1.07)	2.4 (2.45)		
Organisational readiness score	5–9.99	4	13.2 (3.05)	16.4 (2.08)	3.2 (2.17)	0.10	0.73
	10–15	7	16.1 (2.13)	19.8 (1.78)	3.7 (2.32)		

### Effectiveness measure – cleaning performance (UV gel dot audits)

Mixed modelling showed a positive association between pre-intervention bundle alignment and cleaning performance (OR = 1.18; 95% CI: 1.1 to 1.3, Table 3). For a hospital with average alignment (score = 15), the predicted percentage of FTPs cleaned prior to bundle implementation was 48% (95% CI: 43 to 52%).

**Table 3:** Mixed modelling results for the effects of baseline alignment and intervention length on FTP cleaning

Model parameter	Estimate (log OR)	SE	*z*-value	*p*-value
Intercept	−0.10	0.09	−1.04	0.30
Baseline alignment score (per 1 point increase)	0.17	0.04	4.5	< 0.001
Time since start of intervention (per 10 weeks)	0.44	0.01	34.6	< 0.001
Interaction between baseline alignment score and time since start of intervention	−0.01	0.004	−3.0	0.003

*OR* Odds Ratio, *SE* Standard Error

**Fig. 2:**
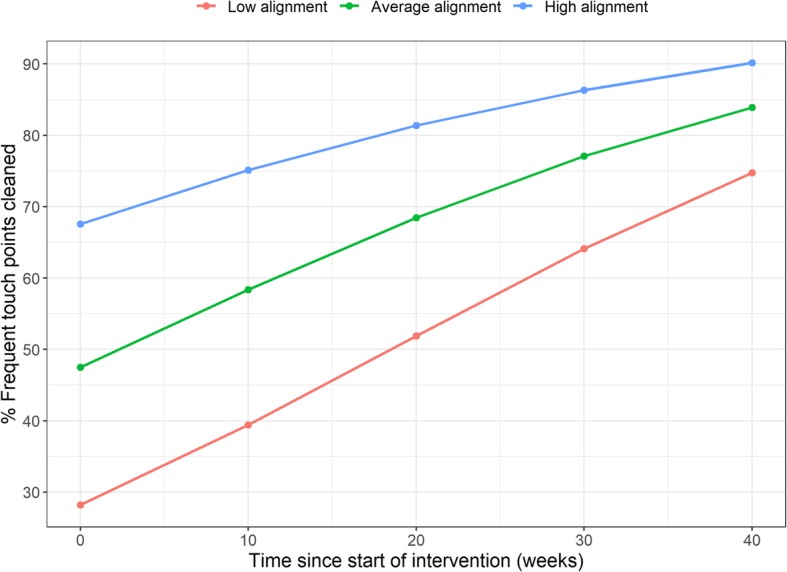
Association between baseline bundle alignment score and cleaning performance as assessed through UV dot audits

Overall, cleaning performance improved during the trial (OR: 1.55; 95% CI: 1.51 to 1.59), however, the estimated interaction effect indicated that the influence of pre-bundle alignment diminished over time (OR = 0.99; 95% CI: 0.98 to 1.0). In the pre-intervention period, the predicted percentage of FTPs cleaned was 28% for hospitals with low alignment (score = 10; 95% CI: 26 to 30%), and 68% for hospitals with high alignment (score = 20; 95% CI: 66 to 70%) (Fig. 2).

By 40 weeks post-implementation, between-group differences had decreased substantially, with cleaning performance ranging from 75% (95% CI: 72 to 77%) to 90% (95% CI: 89 to 92%), for low and high aligning hospitals, respectively.

## Discussion

The REACH trial required structured implementation of a complex, multi-component intervention in 11 different and complex healthcare organisations. This process required both adherence to the study protocol and a pragmatic and responsive tailoring of the cleaning bundle to support local practice change, along with a structured implementation approach.

Overall the implementation strategy worked well. We successfully implemented the bundle and observed improvements in practice, regardless of hospital size, intervention duration and contextual issues such as staff and organisational readiness at baseline (Table 2).

We tailored the implementation and support required for each hospital by carefully quantifying gaps or specific needs before commencement of the trial. For example, four hospitals had gaps for all five bundle components. For these hospitals, implementation plan activities included a strong focus on change mechanisms (such as engagement and communication strategies) and monitoring the practice change required, especially ensuring adequate training and support for gel dot auditing as this was new in all these hospitals.

We identified three hospitals that needed extra support throughout the trial to correctly complete audit activities, so more intensive feedback and support about auditing was provided. In sites with a low score for technique at baseline, trial resources were tailored to the cleaning of the FTPs, with strong uptake of ‘prompt’ style posters: “this week let’s focus on taps” or point of care visual prompts for cleaning teams.

Our study has provided evidence of how difficult it is to implement communication change, reflecting the reality of large hospitals being complex institutions, usually with cleaning staff at the lower end of a strong and culturally-embedded staff hierarchy [17, 21]. Nine of our 11 sites had some gaps in communication practices observed at baseline, and only three of these were able to record improvements in their communication component score. Communication was easier to improve and maintain where there was an opportunity to align with a baseline of effective existing structures and mechanisms; where a hospital had no/low established communication practices it was challenging to introduce, foster and support these. Recognising the challenges of externally based study teams, we created local site teams where possible, working with local change champions and engaging hospital executives early. Communication remained challenging in sites without strong site champions and with weaker executive leadership engagement. Communication was also more difficult to improve at hospitals with dual cleaning workforces, as communication systems across the workforces usually varied.

Due to the study protocol requirements, the study team capacity and the resourcing available at each hospital we needed to balance tailored local facilitation with systematic, multi-site engagement and implementation. We could find no exemplars or implementation templates based on the iPARIHS framework to support a systematic scale-up and roll-out for a multi-site clinical trial. Further, the iPARIHS framework has an underpinning construct of local facilitation as being essential to successful implementation [15], which was not fully operationalised in all trial sites. This is a common issue in large studies where resourcing to support in-depth local facilitation is limited, and alternative approaches must be considered [22].

The toolkit we developed, with templates and our scoring webs, helped address these gaps. Our templates and webs assisted with identifying required practice change and contextual issues prior to implementation. These tools allowed us to assess the relative scale of any issues and to focus behaviour change strategies where they were needed most. In particular this approach helped maintain clarity about the intervention, including fixed and flexible components. Understanding the pragmatics of implementation and knowledge of what worked where and why is important for trial site comparisons, replication and scalability [23]. It is also vital for building an evidence base for policy and practice [24]. A strength of this study, which could be useful in other settings, was the ability to quantify alignment with best practice before and after the bundle was implemented. We were able to assess the extent and fidelity of implementation at each site, factors that are often not documented in clinical trials or large scale quality improvement interventions [25]. The level of fidelity required across diverse healthcare organisations for successful implementation of complex interventions is not well explored or understood [26]. By quantifying the extent of practice change and the impact of this on study outcomes, we were able to confirm that it is possible to reduce the evidence-practice gap in hospital cleaning and the differences in cleaning performance between hospitals over time. The gap between hospitals narrowed over time: those with better alignment scores at baseline started at a higher performance level but across the intervention phase there was little difference between hospitals. The longer the intervention went on, the smaller the gap became.

We showed that fidelity does not have to be perfect to improve intervention outcomes. In fact, most hospitals still had sub-optimal bundle alignment at the end of the study, but the changes were sufficient to demonstrate improvements in cleaning performance and reductions in healthcare associated infection rates [9]. This is a promising message for hospital administrators and environmental cleaning staff, confirming that small evidence-based changes can have a big impact.

## Conclusions

Our research demonstrates that by taking a pragmatic approach - focussing on getting the basics right, and then tailoring implementation efforts - it is possible to improve environmental cleaning in diverse hospital settings. The study results, including reductions in the evidence-practice gap observed between hospitals and improvements in cleaning performance, provide empirical evidence for a mechanism to assist with successful implementation of best practice in infection prevention.

We showed that using a structured framework-based approach to implementation is useful. It allows researchers to balance the priorities of clinical trials with local site needs, providing vital information on the impact and success of the implementation process. This approach could be applied in other pragmatic trials or assist with the implementation of other complex multi-site interventions.

## Supplementary information

**Additional file 1.** Logic model for implementing the REACH bundle

**Additional file 2.** Adapting iPARIHS framework to REACH

**Additional file 3.** Standards for Reporting Implementation Studies (StaRI) checklist

## Data Availability

All relevant data, including non-identifiable participant data and relevant documents such as the study protocol and templates, will be shared upon request, according to the International Committee of Medical Journal Editors (ICMJE) data sharing policy. The gel dots audit data record supporting the conclusions of this article is available in the QUT Research Data Finder repository, 10.25912/5c9c4879d4c99 .

## Abbreviations

CI: Confidence interval
FTPs: Frequent-touch points
HAIs: Healthcare-associated infections
ICMJE: International Committee of Medical Journal Editors
ICU: Intensive Care Unit
i-PARIHS: Integrated Promoting Action on Research Implementation in Health Services
OR: Odds ratio
REACH: Researching Effective Approaches to Cleaning in Hospitals
SD: Standard deviation
SE: Standard error
StaRI: Standards for Reporting Implementation Studies checklist
UV: Ultraviolet
