# Supplementary figures and images for: Effectiveness of a structured, framework-based approach to implementation: the Researching Effective Approaches to Cleaning in Hospitals (REACH) Trial
Source: Antimicrob Resist Infect Control. 2020 Feb 18;9:35. doi: 10.1186/s13756-020-0694-0 (PMC7029491; doi:10.1186/s13756-020-0694-0)

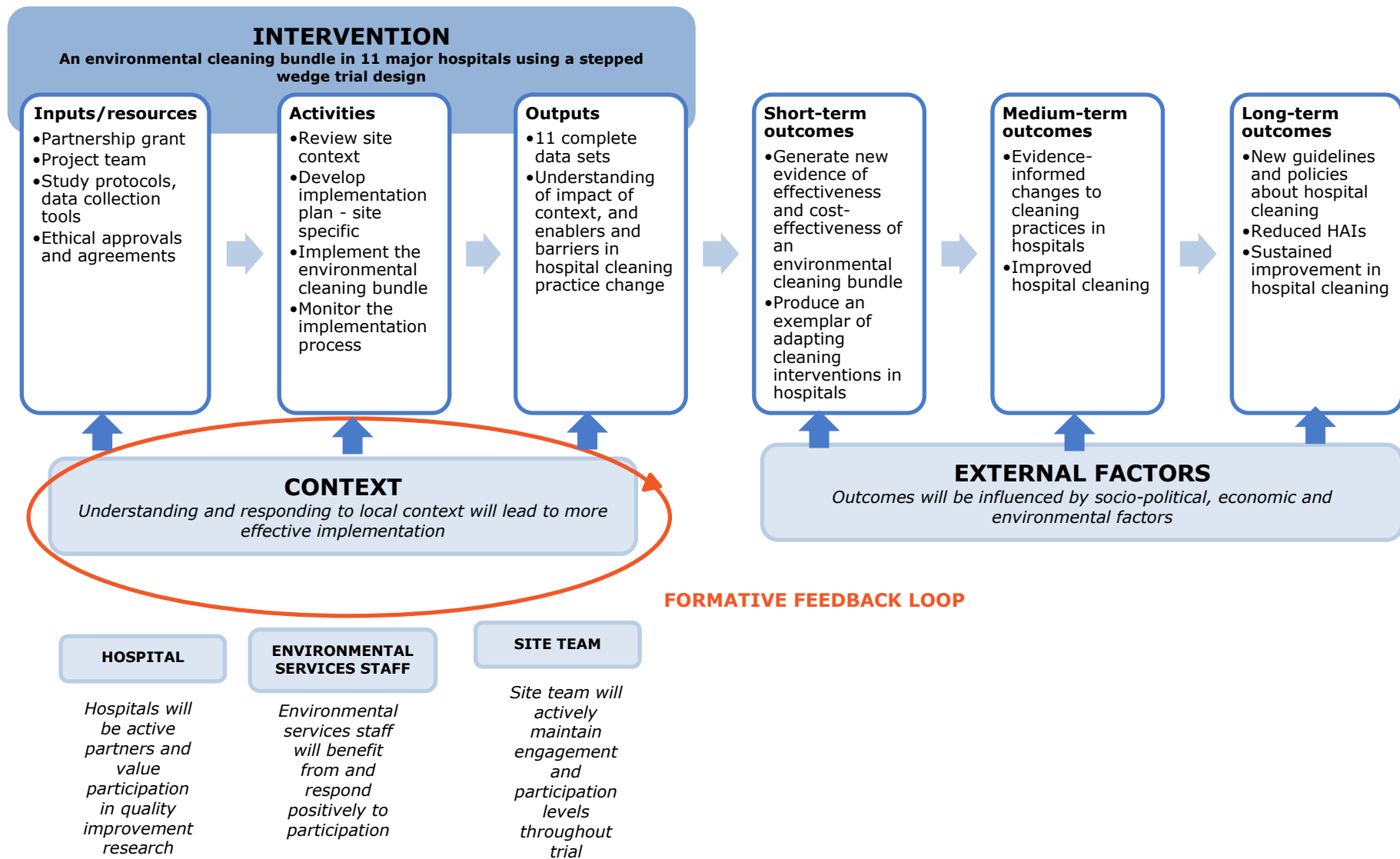

Supplement: Supplementary file 1 — Additional file 1. Logic model for implementing the REACH bundle [file 13756_2020_694_MOESM1_ESM.pdf]
