# Supplementary material for: Effectiveness of a structured, framework-based approach to implementation: the Researching Effective Approaches to Cleaning in Hospitals (REACH) Trial
Source: Antimicrob Resist Infect Control. 2020 Feb 18;9:35. doi: 10.1186/s13756-020-0694-0 (PMC7029491; doi:10.1186/s13756-020-0694-0)
Supplement: Supplementary file 2 — Additional file 2. Adapting iPARIHS framework to REACH [file 13756_2020_694_MOESM2_ESM.pdf]

SUPPLEMENTARY FILE 2

Adapting IPARIHS to REACH

Aim: to determine key context assessment and monitoring questions for the REACH trial

Elements & key questions - iPARIHS	REACH questions	Prompts
**1. Characteristics of the cleaning bundle intervention**		
**Who it affects?**<br>*Who is likely to be affected by the proposed innovation?*	Who is directly impacted?	Composition, roles and responsibilities of site team<br>Environmental services workforce
**Underlying knowledge sources**<br>*Is the evidence for the cleaning bundle viewed by the site as rigorous and robust?*<br>*Is there a shared view about the evidence?*<br>*What other evidence is important at this site?*	How is the bundle perceived?	Is there a shared view about the evidence? What other evidence is important at this site?
**Clarity**<br>*Is the evidence packaged in an accessible and usable form?*<br>*Will people be able to see easily and clearly what is proposed in terms of practice?*		
**Degree of fit (compatibility or contestability)**<br>*How well does it 'fit' the local setting?*<br>*Is it likely to be accepted or contested by those people who have to implement it?*	How does the bundle align with current practice at the site?	What is the extent of change required to implement?<br>*Refer to completed intervention gap analysis*
**Degree of novelty**<br>*How much novelty does the cleaning bundle introduce?*<br>*Will it require significant changes in the processes and/or systems of care delivery?*<br>*Will a knowledge transfer, translation or transformation strategy be required?*		

**Elements & key questions - iPARIHS**	**REACH questions**	**Prompts**
**Likely boundaries**<br>*Will it present a challenge to people's ways of thinking, mental models and relationships?*<br>*What are the implications of this in terms of the likely boundaries that will be encountered?*	What are the potential challenges associated with the bundle at this site?	How will it challenge practice and ES work?<br>What impact could these have?<br>*Refer to completed intervention gap analysis*
**Relative advantage**<br>*Does it offer advantages over the current way of doing things?*<br>*Will it enhance patient /staff experience?*<br>*Could it introduce greater efficiency in the provision of care?*	What are the potential benefits of the bundle at this site?	What advantages are there for cleaning practice, hospital outcomes, team culture<br>How will it improve ES staff work?
**Trialability**<br>*Is there potential to pilot the introduction of the evidence/innovation on a small scale*	N/A	
**2. Recipients**		
**Motivation**<br>*Do individual members of the team want to apply the change in practice? Do individuals see a need to make the change?*<br>*At a collective level, does the team want to apply the change in practice? Do the team see a need to make a change?*	How motivated are ES to change?<br>How does the change align with existing values and opinions?	What are the motivators for ES staff to change?<br>How much do they see a need to change? What is influencing this?<br><br>Consider:<br>individuals<br>team<br>consensus level
**Values & beliefs**<br>*Is the change consistent with individuals' existing values and beliefs?*<br>*Do individuals perceive the proposed change as valuable and worthwhile?*<br>*Is the proposed change seen by the team as valuable and worthwhile?*		
**Clinical consensus**<br>*Is there a shared view or are there differences of opinion e.g. between key individuals or between professional groups /communities of practice?*		

Elements & key questions - iPARIHS	REACH questions	Prompts
**Skills and knowledge**<br>*How well are individual members / team able to implement the proposed change? Do they understand what the change entails? Is it within their current level of knowledge and skills? What additional training and development will be needed?*	What capacity is there to implement the change?	How will staff perceive and approach the change required?<br><br>What specific skills and knowledge will be needed? Consider:<br>individuals, ES team, site team<br>*Refer to analysed surveys*
**Time and resources**<br>*What resources are needed to support the implementation process: time and/or financial support for new skills development, new equipment, expert support and advice? Are these adequate? Are there gaps?*	What resources will be needed?	What are the existing resources?<br>What new/additional resources will be needed?<br>Can these be provided?<br>Consider:<br>Team work/collaboration<br>Existing data/gaps<br>*Refer to analysed surveys*
**Learning environment**<br>*Do environmental services staff understand the modifications needed to routine practice and how to change and embed these?*<br>*Does the team understand the modifications needed to routine practice and how to change and embed these?*		
**Existing data sources**<br>*What data can be used and how to highlight the potential for improvement?*		
**Local opinion leaders**<br>*Which individuals function as local opinion leaders? Will they be supportive or obstructive in terms of introducing the proposed change*	What support will there be for ESW?<br>What human factors will impact the implementation, and how?	Opinion leaders/ champions<br>Collaboration and team cohesiveness<br>*Refer to analysed surveys*
**Collaboration and teamwork**<br>*Is there good inter-professional collaboration and team-work between ES staff and with clinical staff and managers?*<br>*Will support be needed to develop more effective collaboration and team-work?*		

Elements & key questions - iPARIHS	REACH questions	Prompts
**Power & authority**<br>*Do individuals have the necessary authority to carry out the proposed changes?*<br>*Have key individuals whose support is needed been identified?*<br>*Are they engaged in discussing and planning implementation?*<br>*Does the team have the necessary authority to carry out the proposed changes?*		
**Professional boundaries & networks**<br>*Are the potential barriers to implementation known? Are there strategies in place to address these?*		
**3a. Inner context - local level**		
**Formal & informal leadership support**<br>*Who are the formal and informal leaders at a local level?*<br>*Are the leaders helping to create a facilitative context through providing motivation and support, creating a vision and reinforcing the change process?*<br>*Is there a distributed and devolved style of management?*	What aspects of the ES and IP&C culture support innovation and change?<br><br>Who are the facilitators and how do they provide support?<br><br>What processes and practices support change, and how?<br><br>What are the barriers in relation to team culture?	Who are the local supports?<br>How involved are ESW in initiating change?<br><br>What is the past experience of introducing changes at a team level?<br><br>*Refer to analysed surveys*
**Culture**<br>*Is there a culture that supports innovation and change?*<br>*Do staff feel actively involved in decisions that affect them?*<br>*Are staff trusted to introduce new ideas into practice? Do staff and patients feel valued?*		
**Past experience of change**<br>*What is the past experience of introducing changes at a local level?*		
**Mechanisms for embedding change / Learning, evaluation & feedback processes**<br>*Are there mechanisms in place to support learning and evaluation and to embed changes in routine practice e.g. regular team meetings, audit and feedback processes,*		

**Elements & key questions - iPARIHS**	**REACH questions**	**Prompts**
professional development opportunities and performance review systems?		
**3b. Inner context - organisational level**		
**Organisational priorities**<br>*Do the evidence/innovation and the changes proposed align with the strategic priorities for the organization?*	How does the trial align with strategic priorities at the hospital?	Are there any competing priorities or agendas?
**Leadership & senior management support**<br>*Has the support of key individuals and leaders within the organization been sought and secured?*	What level of support is there from the hospital executive?	How engaged are the hospital executive?<br>Who are they?
**Culture / history of innovation and change**<br>*Is there a culture that supports innovation and change?*<br>*Is there a history of successful and sustained change within the organization?*<br>*Do the senior management team actively seek opportunities for improvement and encourage ideas and feedback from patients, the public and staff?*	What approaches and systems support innovation and change?	What is the past experience of introducing changes at the hospital?<br>What processes and practices support change, and how?<br>How is change sustained/ embedded?<br>What are the barriers?
**Systems & processes /Absorptive capacity**<br>*Does the organization have systems and processes in place that support innovation and change e.g. effective information and communication systems, opportunities for networking and learning across departments/teams?*<br>*Are there mechanisms in place for embedding changes in routine practice e.g. formal policies and procedures?*		
**4. Outer context**		

Elements & key questions - iPARIHS	REACH questions	Prompts
**Policy drivers & priorities**<br>*Do the evidence/innovation and the changes proposed align with the strategic priorities for the wider health system e.g. in terms of current health policy, national priorities for action and improvement?*<br>*Is this likely to influence the implementation project?*	What aspects of the external context will most influence the trial implementation, and how?	What external motivators and incentives exist?<br>Are there competing agendas?<br>How stable is the wider context?<br>What is the likely impact from regulatory frameworks (accreditation, government) and external networks?
**Incentives & mandates**<br>*Are there incentives in the wider health system that reinforce the proposed change e.g. pay for performance schemes, regulatory requirements etc.?*		
**Regulatory frameworks**<br>*What are the key regulatory frameworks?*		
**Environmental (in)stability**<br>*How much stability/instability is there in the wider health system?*		
**Inter-organisational networks & relationships**<br>*Are there inter-organizational networks (e.g. specialised clinical networks) that will be helpful in terms of supporting the proposed changes?*	What opportunities are there for engaging with and leveraging external support?	
